# Pathways for Modulating Exosome Lipids Identified By High-Density Lipoprotein-Like Nanoparticle Binding to Scavenger Receptor Type B-1

**DOI:** 10.1038/srep22915

**Published:** 2016-03-11

**Authors:** Nicholas L. Angeloni, Kaylin M. McMahon, Suchitra Swaminathan, Michael P. Plebanek, Iman Osman, Olga V. Volpert, C. Shad Thaxton

**Affiliations:** 1 Department of Urology, Northwestern University Feinberg School of Medicine, Chicago, IL, United States; 2 Simpson Querrey Institute for BioNanotechnology, Northwestern University Feinberg School of Medicine, Chicago, IL United States; 3 Robert H. Lurie Comprehensive Cancer Center, Northwestern University Feinberg School of Medicine, Chicago, IL, United States; 4 Driskill Graduate Program in Life Sciences, Northwestern University, Chicago, IL, United States; 5 The Ronald O. Perelman Department of Dermatology, New York University School of Medicine, New York, NY, United States; 6 Laura and Isaac Perlmutter Cancer Center, New York University, Langone Medical Center, New York, NY, United States; 7 International Institute for Nanotechnology (IIN), Northwestern University, Evanston, IL, United States

## Abstract

Exosomes are produced by cells to mediate intercellular communication, and have been shown to perpetuate diseases, including cancer. New tools are needed to understand exosome biology, detect exosomes from specific cell types in complex biological media, and to modify exosomes. Our data demonstrate a cellular pathway whereby membrane-bound scavenger receptor type B-1 (SR-B1) in parent cells becomes incorporated into exosomes. We tailored synthetic HDL-like nanoparticles (HDL NP), high-affinity ligands for SR-B1, to carry a fluorescently labeled phospholipid. Data show SR-B1-dependent transfer of the fluorescent phospholipid from HDL NPs to exosomes. Modified exosomes are stable in serum and can be directly detected using flow cytometry. As proof-of-concept, human serum exosomes were found to express SR-B1, and HDL NPs can be used to label and isolate them. Ultimately, we discovered a natural cellular pathway and nanoparticle-receptor pair that enables exosome modulation, detection, and isolation.

Exosomes are nanovesicles between 30–150 nm in diameter ubiquitously secreted by eukaryotic cells. Exosomes contain a variety of nucleic acids, protein, and lipids representative of the parent cell^1^. Enhanced exosome production is implicated in the pathogenesis of human diseases, like cancer^2^,^3^. Importantly, exosomes, including tumor-derived exosomes, deliver cargo between different cell types and specifically target cancer, endothelial, and immune cells^3^,^4^,^5^,^6^ to enhance primary tumor development and facilitate metastasis^2^,^3^,^6^. Because of their role in disease progression and inherent targeting properties there is significant interest in exosome production, detection, and manipulation of their molecular content^7^,^8^. For example, isolated cells have been engineered to overexpress certain potentially therapeutic cargoes that can be packaged into secreted exosomes whereupon the exosomes are then systemically delivered as a therapeutic^9^. In addition, dendritic cells have been shown to process antigens *ex vivo* that are then presented on the surface of secreted exosomes, which can then be isolated and systemically delivered to stimulate favorable anti-tumor immune responses^10^. Also, exosomes can first be isolated, and then tailored to incorporate certain therapeutic or enhanced targeting molecules using a number of techniques^9^. However, these methods have significant limitations for *in vivo* applications and variability with regard to cargo loading^9^. We hypothesized that targeting a cell surface receptor present on both parent cells and exosomes with a tailorable, synthetic ligand would identify a natural pathway of exosome production and a way to modulate exosome cargo.

Recent studies demonstrate scavenger receptor type B-1 (SR-B1) is expressed by multiple cancer cells and their exosomes, including melanoma and prostate cancer^11^,^12^. Scavenger receptor type B-1 (SR-B1) is a high-affinity receptor for high-density lipoproteins (HDL). SR-B1 is known to mediate the transfer of phospholipids between HDL and the cell membrane^13^. We reported and characterized a synthetic HDL nanoparticle (HDL NP) platform^14^,^15^,^16^, whereby HDL NPs bind SR-B1^17^,^18^,^19^ and can be tailored to incorporate a wide variety of molecules such as phospholipids, nucleic acids, and proteins^17^,^18^,^19^,^20^,^21^. Accordingly, we modified HDL NPs to contain fluorescently labeled phospholipids in order to identify SR-B1 as a critical cell surface receptor that enables manipulation of exosome content via a natural cellular pathway. Data show the successful synthesis of an HDL NP that carries a rhodamine labeled phospholipid (Rh-HDL NP). In an SR-B1 dependent manner, Rh-HDL NPs engage parent cells, transfer through the known sequence of intracellular exosome production events, leading to the production of rhodamine labeled exosomes. The fluorescent exosomes can be detected using direct flow cytometry, a method that does not require complex pre-processing steps, and the exosomes are stable in human serum. Additionally, SR-B1 is present in exosomes obtained from the serum of patients with melanoma, which allows for labeling and isolation of the exosomes using Rh-HDL NP.

## Results

### Synthesis of Rh-HDL NPs

We synthesized HDL NPs with rhodamine-labeled fluorescent phospholipids (Rh-HDL NP, see Methods). Data demonstrate that there are ~17 fluorescent phospholipids on each Rh-HDL NP (Supplementary Table S1). Also, incorporation of the rhodamine fluorophore was evident by a noticeable absorption peak at 560 nm (Supplementary Fig. S1). The Rh-HDL NPs have physical properties similar to those of HDL NPs that lack the labeled phospholipid (Supplementary Table S1).

### Characterization of Rh-HDL NP containing exosomes

**Figure 1:**
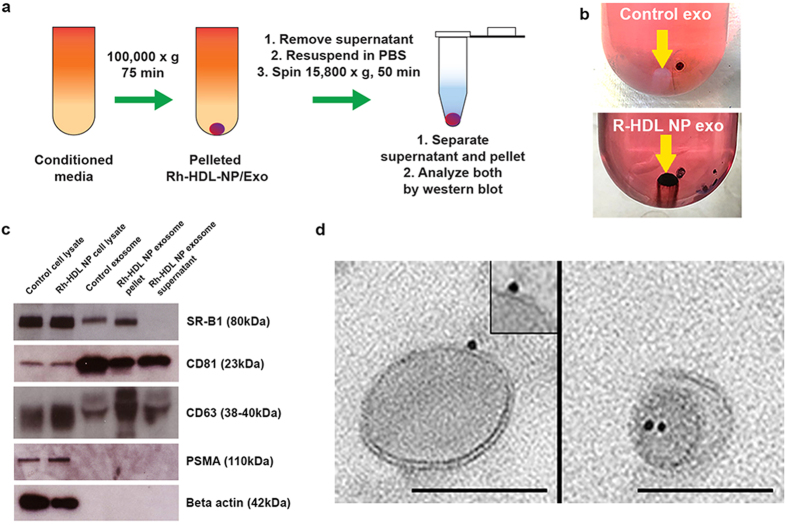
SR-B1 expression in CWR22Rv1 cells and exosomes and Rh-HDL NP association. (**a**) Scheme demonstrating isolation method for exosomes from untreated and Rh-HDL NP treated cells. (**b**) Exosome pellet following ultracentrifugation of conditioned media from untreated cells (top) and cells treated with Rh-HDL NPs (bottom). (**c**) Western blot of cell lysates and exosomes from untreated and Rh-HDL NP treated CWR22Rv1 cells. (**d**) TEM of exosomes from cells treated with Rh-HDL NP (49,000×; inset 98,000×). Scale bar: 100 nm.

We initiated our studies with CWR22Rv1 prostate cancer cells. Cells were treated for 96 hours with Rh-HDL NPs (20 nM) or left untreated (control). Uptake of Rh-HDL NPs by CWR22Rv1 cells was confirmed by flow cytometry (Supplementary Fig. S2). Exosomes were then isolated from conditioned media by ultracentrifugation (Fig. 1a). The exosomes from Rh-HDL NP-treated cells contained gold nanoparticles, as indicated by the darkened exosome pellet (Fig. 1b). Western blotting confirmed SR-B1 expression by the cells and their exosomes (Fig. 1c). Tetraspanins, such as cluster of differentiation 63 (CD63) and 81 (CD81), are proteins enriched in exosomes^22^,^23^,^24^ and are commonly used as exosome markers^24^. We used each of these as positive control markers for exosomes (Fig. 1c). The addition of Rh-HDL NPs did not change SR-B1 expression (Fig. 1c) or exosome production by the parent cells (Supplementary Fig. S3). In order to ascertain the association between the Rh-HDL NPs and exosomes obtained by ultracentrifugation, the pellet was re-suspended and then spun at a lower speed capable of pelleting the Rh-HDL NPs, and therefore Rh-HDL NP-associated exosomes, but not exosomes free of the Rh-HDL NPs (Fig. 1a). Western blot analysis was then performed on the pellet and supernatant. Data show that CD81 and CD63 are enriched in both the pellet and the supernatant (Fig. 1c) suggesting that Rh-HDL NP associated with a sub-population of exosomes. Interestingly, SR-B1 was only found in the pellet fraction suggesting that the Rh-HDL NPs are selectively sorted to SR-B1 positive exosomes (Fig. 1c). Of note, prostate specific membrane antigen (PSMA), a common cell surface marker with specificity to prostate cancer cells, was only detectable in cell lysates, regardless of Rh-HDL NP treatment (Fig. 1c) and was not detected in the exosome pellet or supernatant (Fig. 1c). The lack of beta actin confirmed that exosome preparations were free from cellular debris (Fig. 1c). To further characterize the association between Rh-HDL NPs and exosomes, we performed transmission electron microscopy (TEM), dynamic light scattering (DLS), and nano-tracking analysis (NTA) on isolated exosomes. TEM imaging revealed HDL NPs associated with the bilayer of exosomes (Fig. 1d). The measured size of isolated exosomes was not significantly changed by Rh-HDL NP treatment via DLS and NTA (Supplementary Fig. S4). Taken together, these data demonstrate that Rh-HDL NPs are incorporated in SR-B1-positive exosomes produced by CWR22Rv1 cells.

**Figure 2:**
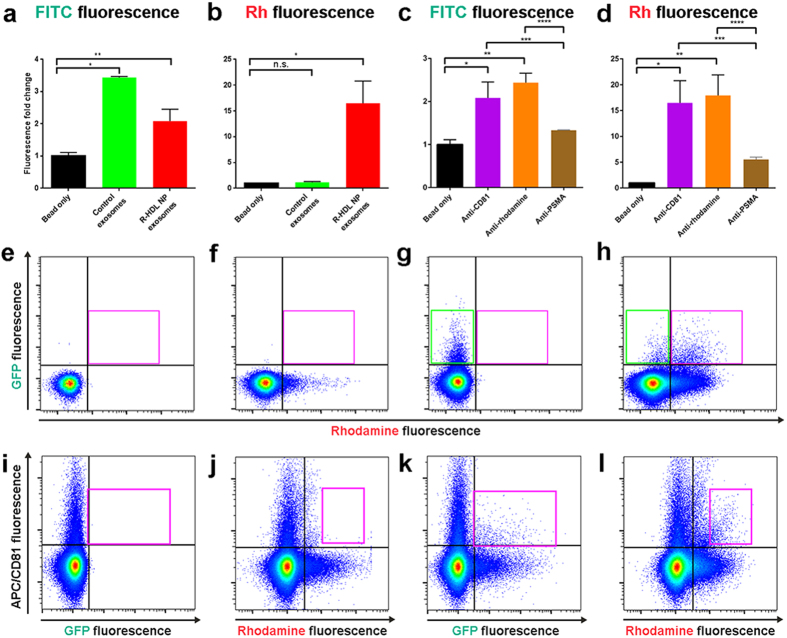
Detection of CWR22Rv1 cell exosomes using flow cytometry. (**a**) Quantification of FITC (total exosome) fluorescence using anti-CD81 ExoFlow beads incubated with exosomes from control (green bar) or Rh-HDL NP (red bar) treated cells. *(P < 0.001), **(P = 0.03). (**b**) Rhodamine fluorescence was quantified on the same set of beads used in (**a**). *p = 0.0182. (**c**) ExoFITC fluorescence was measured after anti-rhodamine beads were utilized to isolate Rh-HDL NP exosomes. *(P = 0.029), **(P = 0.007), ***(P = 0.05), ****(P = 0.001). (**d**) The same exosomes as in (**c**) were analyzed for rhodamine fluorescence. *(P = 0.018), **(P = 0.013), ***(P = 0.034), ****(P = 0.024). A two-tailed t-test was used to determine significance. (**e-l**) Direct flow cytometry of: (**e**) Filtered PBS, (**f**) Rh-HDL NP, (**g**) CWR22Rv1-GFP exosomes (green box), (**h**) Rh-HDL NP exosomes (pink box), (**i**) APC anti-CD81 antibody alone (control) (**j**) CD81 antibody and Rh-HDL NP (control) (**k**) CD81^+^/GFP^+^ exosomes (pink box), and (**l**) CD81^+^/Rh^+^ exosomes (pink box).

Having determined that the Rh-HDL NPs associate with exosomes, we next investigated whether the exosomes isolated from conditioned media of CWR22Rv1 cells exhibited rhodamine fluorescence. Initially, we utilized conventional flow cytometry techniques by employing the commercially available ExoFlow kit. The kit contains magnetic beads coated with antibodies against CD81, capable of binding free exosomes. Exosomes isolated by ultracentrifugation were incubated with the beads, and the captured exosomes were stained with an exosome-specific fluorescein isothiocyanate (FITC) dye, included with the kit. The fluorescent signal from the beads was analyzed using flow cytometry. Based upon the FITC signal, exosomes were detected from both control and Rh-HDL NP treated cells (Fig. 2a). Using the same sets of beads, rhodamine (Rh) fluorescence was analyzed, and data show a nearly 15-fold increase in Rh fluorescence from Rh-HDL NP exosomes as compared to the untreated controls (Fig. 2b), demonstrating effective incorporation of the rhodamine-labeled phospholipid into the exosomes. We then utilized beads coated with an anti-rhodamine antibody. We observed similar fluorescent recovery from Rh-HDL NP exosomes as measured either by FITC (Fig. 2c) or Rh (Fig. 2d). Importantly, these data also demonstrate that the rhodamine phospholipid was available for antibody binding on the surface of the exosomes, which provides data consistent with the expected orientation of the exosome membrane with reference to Rh-HDL NP binding SR-B1 on the cell membrane (Fig. 1d and *vide infra*). As a negative control, anti-PSMA beads were minimally effective in retrieving Rh-HDL NP exosomes as measured by FITC (Fig. 2c) and Rh (Fig. 2d). These data demonstrate specific retrieval of fluorescent exosomes using either exosome (CD81) or Rh-HDL NP (rhodamine) specific markers.

### Direct flow cytometry of Rh-HDL NP containing exosomes

While bead-based flow cytometry analysis is a common tool for exosome characterization, direct measurement of exosomes using flow cytometry is an emerging technique^25^ that enables rapid exosome profiling with minimal processing. Toward this end, we custom-calibrated an LSRFortessa analyzer with low noise electronics to detect particles in the exosome size range (Supplementary Fig. S5, <200 nm). Using this setup we compared exosomes isolated by ultracentrifugation from green fluorescent protein (GFP) expressing CWR22Rv1 cells with or without Rh-HDL NP treatment. The incorporation of GFP into the exosomes provides a control for exosome detection. Phosphate buffered saline (PBS) (Fig. 2e) and Rh-HDL NPs (Fig. 2f) were used as negative and rhodamine positive (Rh^+^) controls, respectively. Flow cytometry demonstrated a pronounced GFP-positive (GFP^+^) population in the untreated exosome sample without detectable Rh^+^ fluorescence (Fig. 2g, green box). Rh-HDL NP treated cells resulted in a GFP^+^/Rh^+^ population (Fig. 2h, pink box). To further demonstrate the identity of the labeled structures as exosomes, the same samples were analyzed for the presence of CD81 using an allophycocyanin (APC)-tagged anti-CD81 antibody (CD81^+^). Signal from the CD81 antibody can be detected (Fig. 2i), and data show the CD81 antibody did not label the Rh-HDL NP (Fig. 2j). Adding the CD81 antibody to GFP^+^ exosomes reveals a CD81^+^/GFP^+^ population (Fig. 2k, pink box). Exosomes from Rh-HDL NP treated cells are shown to be CD81^+^/Rh^+^ (Fig. 2l, pink box). For further confirmation, we repeated these experiments using an APC-tagged anti-CD63 antibody. Similar results were obtained (Supplementary Fig. S6). These data confirm the GFP^+^/Rh^+^ population is exosomes, and that direct flow cytometry can be used for detection.

### SR-B1 is required for Rh-HDL NP loading of exosomes

**Figure 3:**
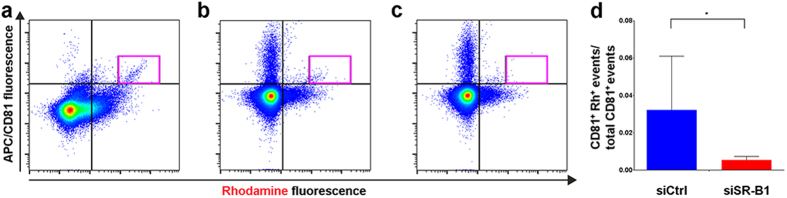
Knockdown of SR-B1 in A375 melanoma cells reduces Rh-HDL NP labeling of exosomes. (**a**) CD81^+^/Rh^+^ exosomes (pink gate) isolated from GFP-SR-B1 A375 conditioned media by ultracentrifugation. (**b**) CD81^+^/Rh^+^ exosomes (pink gate) in conditioned media from GFP-SR-B1 A375 cells treated with control siRNA. (**c**) CD81^+^/Rh^+^ exosomes (pink gate) in conditioned media from GFP-SR-B1 A375 cells treated with siRNA to SR-B1. (**d**) Quantification of CD81^+^/Rh^+^ events normalized to total CD81^+^ events in control and anti-SR-B1 siRNA treated cells (n = 4/group). Statistical significance determined using Mann-Whitney U test. *(P = 0.0286).

To confirm that SR-B1 is required for Rh-HDL NP binding to exosomes, and to demonstrate the broader applicability of the technology, we used the SR-B1 positive melanoma cell line, A375, which is clearly engaged by Rh-HDL NP (Supplementary Fig. S7). These cells were transfected with a plasmid encoding GFP-tagged SR-B1 (GFP-SR-B1). Expression of wild type and GFP-SR-B1 in cells and exosomes was not altered by Rh-HDL NP treatment (Supplementary Fig. S8). Exosomes isolated by ultracentrifugation were analyzed by direct flow cytometry. Exosomes from wild type SR-B1 expressing cells were not GFP^+^ as compared to those from A375 cells expressing GFP-SR-B1 (Supplementary Fig. S8). We next utilized the CD81 antibody to detect CD81^+^ exosomes from GFP-SR-B1 cells treated with Rh-HDL NPs. Flow cytometry revealed a clear CD81^+^/Rh^+^ population (Fig. 3a, pink gate) demonstrating labeling of melanoma exosomes by Rh-HDL NPs. Surprisingly, there is a minimal CD81^+^/GFP^+^ population despite the presence of a GFP^+^/Rh^+^ population (Supplementary Fig. S8). Together, these data suggest that CD81^+^/Rh^+^ exosomes result from Rh-HDL NP association with wild-type SR-B1. To further explore the identity of the GFP^+^/Rh^+^ population of exosomes, we employed the CD63 antibody to stain isolated exosomes. Data reveal populations that are CD63^+^/GFP^+^ and CD63^+^/Rh^+^ (Supplementary Fig. S8), likely representing exosomes. These data highlight uncertainties of efficient and exclusive exosome loading of modified proteins using plasmid or viral vectors and the heterogeneity of exosome populations. Next, to implicate SR-B1, we performed SR-B1 knockdown experiments using a targeted siRNA and confirmed target knockdown by western blot and flow cytometry (see Supplementary Fig. S9) For these experiments, conditioned media from treated cells was subject to direct flow cytometry to demonstrate that exosome isolation by ultracentrifugation was not necessary. Of note, direct flow cytometry results (Fig. 3b) are qualitatively similar to those obtained after exosome purification via ultracentrifugation (Fig. 3a). SR-B1 specific siRNA caused a significant reduction in the CD81^+^/Rh^+^ population (Fig. 3c, pink gate) as compared to cells treated with non-silencing control siRNA (Fig. 3b, pink gate), and quantification of the ratio between CD81^+^/Rh^+^ to total CD81^+^ events is shown in Fig. 3d. Adding the Rh-HDL NP to growth medium alone does not result in CD81^+^/Rh^+^ events (Supplementary Fig. S9). Overall, results show that Rh-HDL NPs are incorporated into exosomes in an SR-B1 dependent manner.

### Internalization of Rh-HDL NP by target cells

**Figure 4:**
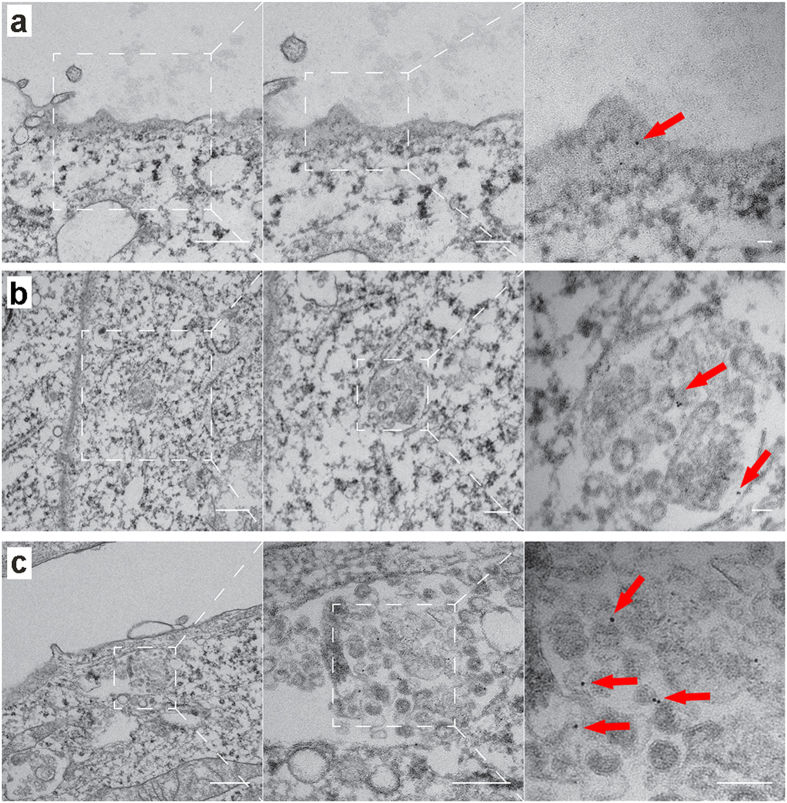
Visualization of Rh-HDL NPs after addition to cells by TEM. (**a**) Rh-HDL NP on the cell membrane at 2 hours (red arrows). Left to right: 18,500×, 30,000×, 68,000×. (**b**) Rh-HDL NP on the inner membrane of an early endosome-like structure at 6 hours (red arrows). Left to right: 11,000×, 23,000×, 68,000×. (**c**) Rh-HDL NP on the outer membrane of microvesicles within a multivesicular body-like structure at 6 hours (red arrows). Left to right: 13,000×, 23,000×, 49,000×. For each panel, scale bars are 500 nm, 200 nm, and 50 nm from left to right.

Data showing that knockdown of SR-B1 reduced Rh^+^ exosomes suggests that one possibility for labeling exosomes requires binding of SR-B1 on the cell membrane and incorporation of the receptor ligand complex into exosomes through an intracellular pathway. The presence of the gold nanoparticle in the core of Rh-HDL NPs provided a unique opportunity to visualize the nanoparticles using TEM and corresponding sub-cellular anatomy. To this end, we treated A375 melanoma cells with Rh-HDL NPs and examined them at 2, 6, and 16 hours following treatment. Strikingly, images reveal clear binding to the cell membrane (Fig. 4a) and internalization into early endosomes (Fig. 4b) and structures that resemble the multivesicular body (MVB) (Fig. 4c). The MVBs have vesicles that resemble exosomes, some of which have gold nanoparticles associated with their membrane. Also, exosomes found outside of the cells are clearly associated with gold nanoparticles (Fig. 4d). Consistent with our model (*vide infra*), gold nanoparticles are near exclusively located to the inner membrane of early endosomes or the outer membrane of exosomes. Ultimately, these data provide direct evidence of an intracellular thoroughfare where Rh-HDL NPs engage cell surface SR-B1. Both the Rh-HDL NP and SR-B1 are transported through the cell for eventual release on exosomes.

### Rh-HDL NP containing exosomes are stable in human serum

**Figure 5:**
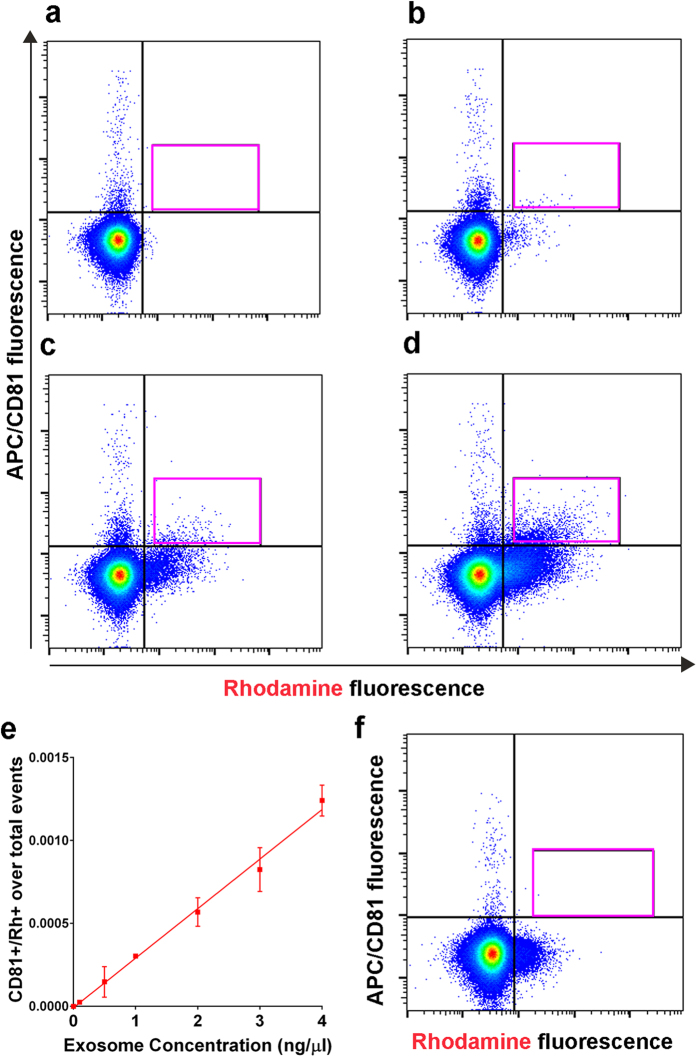
Detection of Rh-HDL NP labeled exosomes spiked into human serum. Direct flow cytometry of human serum incubated with anti-CD81 antibody. (**a**) Anti-CD81 antibody alone. Exosomes from A375 cells after Rh-HDL NP treatment at (**b**) 0.1 ng/μL, (**c**) 1 ng/μL, and (**d**) 4 ng/μL after a 24-hour incubation. (**e**) Quantification of CD81^+^/Rh^+^ events normalized to total events (n = 3/point, r^2^ = 0.9778). (**f**) Rh-HDL NPs incubated in serum from a healthy individual and stained with anti-CD81 antibody.

To establish the potential implications for this technology for *in vivo* applications, we determined the stability of Rh-HDL NP labeled exosomes in human serum. We obtained blood samples from healthy volunteers, added Rh-HDL NP labeled A375 exosomes in increasing concentrations, isolated the serum component, stained for CD81, and then analyzed the samples via flow cytometry immediately and 24 hours following exosome addition. Serum alone was not Rh^+^ (Supplementary Fig. S10, 0 hrs, and Fig. 5a, 24 hrs). Increasing concentrations of CD81^+^/Rh^+^ exosomes were detected at the immediate time point (Supplementary Fig. S10) and at 24 hours (Fig. 5b–d). The number of CD81^+^/Rh^+^ events linearly correlated with exosome concentration at the immediate (Supplementary Fig. S10) and 24 hour time points (Fig. 5e). Thus, tailored exosomes maintain stability in serum and can be detected via Rh^+^ fluorescence. Furthermore, Rh-HDL NPs added to human serum reveal that there is not significant background due to binding of native exosomes after the 24-hour incubation period (Fig. 5f). Collectively, these data show that Rh-HDL NP labeled exosomes are stable in serum for prolonged periods and can be detected using direct flow cytometry.

### Rh-HDL NP detection and isolation of free exosomes that express SR-B1

**Figure 6:**
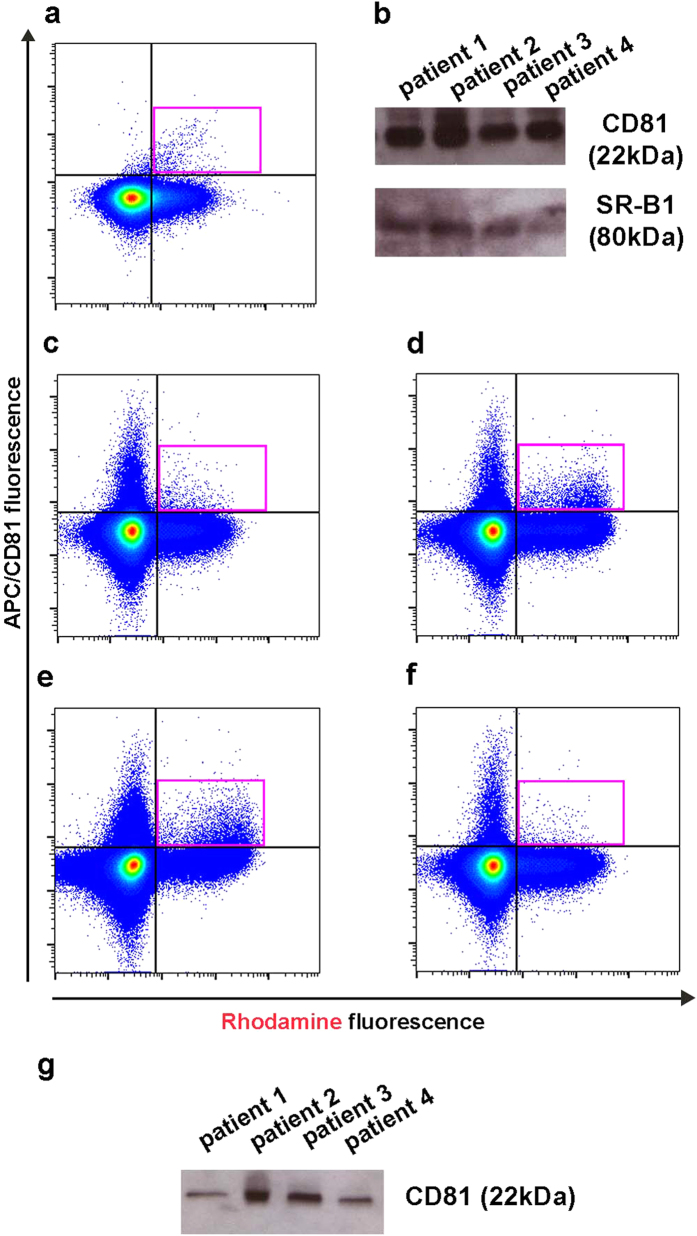
Detection of Rh^+^ exosomes in serum from human patients with melanoma. (**a)** Flow cytometry of A375 exosomes isolated from untreated cells then incubated with Rh-HDL NP and stained with APC anti-CD81. (**b**) Western blot of isolated serum exosomes from human patients with melanoma. (**c–f**) Flow cytometry of serum from human melanoma patients incubated with Rh-HDL NP. Plots are in the same order as loaded on the western blots in (**b,g**). (**g**) Western analysis of Rh-HDL NPs isolated following incubation with human serum samples.

To this point, data have been collected after treating cultured cells with Rh-HDL NPs and subsequently isolating exosomes. As exosomes contain SR-B1, we hypothesized that HDL NPs could bind free exosomes. Toward this end, we added Rh-HDL NPs to purified A375 exosomes and performed flow cytometry staining for CD81. Data revealed a CD81^+^/Rh^+^ population of exosomes confirming that the Rh-HDL NPs can bind SR-B1 on free exosomes (Fig. 6a). In proof-of-concept studies to demonstrate potential diagnostic utility of SR-B1 expression and HDL NPs, we measured CD81 and SR-B1 in exosomes isolated from the serum of patients diagnosed with melanoma. Our (Supplementary Fig. S8) and published data^12^ show that SR-B1 is found in exosomes isolated from cultured melanoma cells. We isolated exosomes from serum samples and performed Western blot for CD81 and SR-B1. Data show that CD81 and SR-B1 (Fig. 6b) are present in exosomes from patients diagnosed with melanoma. Further, serum from each patient was incubated with Rh-HDL NPs and stained with CD81 antibody and then subjected to direct flow cytometry. Data reveal populations that are CD81^+^/Rh^+^ (Fig. 6c–f). Finally, serum samples incubated with Rh-HDL NPs were subjected to standard centrifugation (15,800 × *g*) to pellet the gold nanoparticles, presumably, along with the bound exosomes. Western blot of the resulting pellet showed the presence of CD81 (Fig. 6g). Ultimately, Rh-HDL NPs may be useful for binding, detecting, and isolating SR-B1 exosomes present in human serum.

### Cellular uptake of exosome and HDL NP lipid cargo

**Figure 7:**
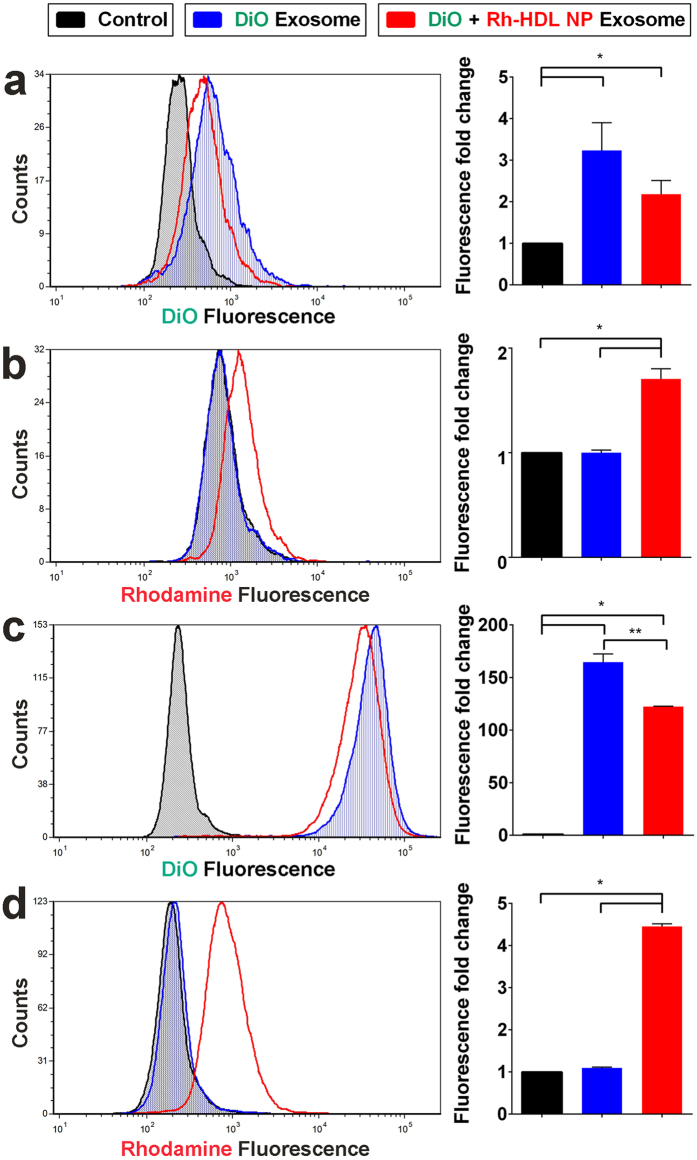
Cellular uptake of Rh-HDL NP labeled exosomes. (**a**) DiO fluorescence of A375 cells after 2 hours of exosome treatment. *(P ≤ 0.0427) (**b**) Rhodamine fluorescence of A375 cells after 2 hours of exosome treatment. *(P = 0.0094) (**c**) DiO fluorescence of A375 cells after 24 hours of exosome treatment. *(P < 0.005), **(P = 0.0161) (**d**) Rhodamine fluorescence of A375 cells after 24 hours of exosome treatment. *(P = 0.002). n = 3 each sample. Statistical significance was determined by a two-tailed t-test. Error bars are ± standard deviation.

Prior work from our group demonstrates that HDL NPs bind cellular SR-B1 and modulate the uptake of exosomes^26^. Thus, we investigated if Rh-HDL NPs bound to free exosomes prevented exosome uptake. Free A375 exosomes were labeled with a lipophilic intercalating dye (DiO), washed, and then treated with Rh-HDL NPs. We confirmed the identity of DiO-labeled exosomes by both CD81 and CD63 staining (Supplementary Fig. S11). Untreated A375 cells were exposed to DiO-labeled exosomes or DiO-labeled exosomes treated with Rh-HDL NPs. Flow cytometry data collected after incubating for two or twenty-four hours revealed clear uptake of DiO labeled exosomes and Rh-HDL NP treated DiO exosomes (Fig. 7). Of note, the slight reduction in exosome uptake observed in the Rh-HDL NP cases likely resulted from a small amount of residual free Rh-HDL NPs and is consistent with our previous findings.

## Discussion

**Figure 8:**
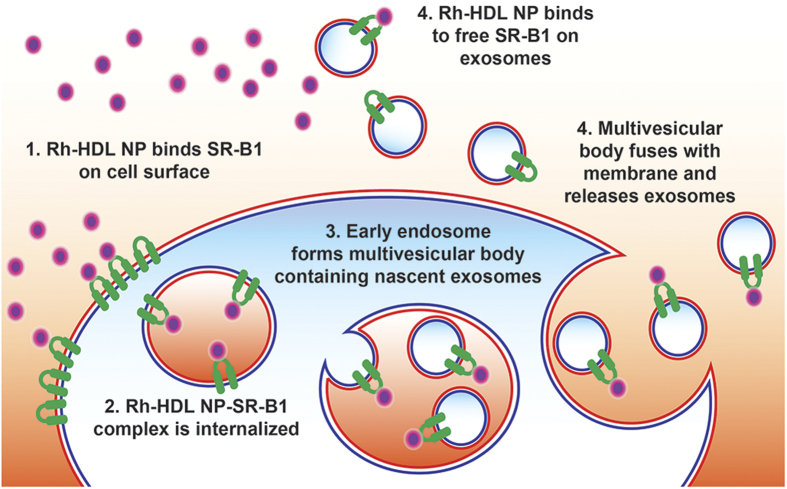
Scheme for Rh-HDL NP binding and exosome labeling. Rh-HDL NPs may label exosomes by two pathways: binding of SR-B1 on the cell surface resulting in internalization and incorporation into exosomes at time of synthesis or binding of SR-B1 in free exosomes after secretion from the parent cell.

Identifying and targeting a natural cellular pathway of exosome production provides a new mechanism for efficient and stable manipulation of exosomes that may enable *in vivo* applications. Rh-HDL NPs bind SR-B1 on parent cells and then become incorporated into newly formed exosomes. This synthetic ligand/receptor pair takes advantage of an inherent thoroughfare between the parent cell membrane and exosomes, with SR-B1 playing a crucial role. Our data support a model (Fig. 8) whereby HDL NPs bind to cell surface SR-B1, which remain associated through subsequent exosome formation. As exosomes maintain the same membrane polarity as parent cells^1^, it is unlikely labeling takes place via other uptake methods since the surface of the developing exosomes does not contact the cytoplasm. The exosome TEM data (Fig. 4a–d) and capture of intact exosomes by anti-rhodamine antibody (Fig. 2c,d) support this model. In addition, Rh-HDL NPs can bind SR-B1 in free exosomes, which may provide subsequent opportunities for exosome detection and enrichment.

Interestingly, SR-B1 has been shown to associate with CD81^27^,^28^. The detection of GFP^+^/Rh^+^ and CD63^+^/GFP^+^ events in A375 melanoma cells expressing GFP-SR-B1, but not CD81^+^/GFP^+^ events, suggests that GFP modification of SR-B1 may prevent GFP-SR-B1 sorting to CD81^+^ exosomes. Further work is required to more fully understand cargo loading and manipulation of heterogeneous populations of exosomes.

Finally, we developed novel parameters for exosome characterization by flow cytometry which require minimal processing, and is time and cost efficient. Direct analysis of microvesicles by flow cytometry is an emerging field, and there is still much work required to develop instrumentation capable of microparticle sorting and standard techniques for routine use^25^. However, our data presented here shows much promise for rapid analysis of exosomes by flow cytometry. Further study of a greater number of patients with SR-B1-expressing tumors is required to determine if SR-B1 is a useful biomarker.

## Methods

### Human Subject Use

Experiments involving humans were approved by the Northwestern University Institutional Review Board (IRB, protocol number STU00200368-MOD0001) and the New York University School of Medicine IRB (protocol number i10362) and carried out in accordance with approved guidelines. Informed consent was obtained from all those enrolled and melanoma patient samples were completely de-identified before transfer to Northwestern.

### Rhodamine HDL NP (Rh-HDL NP) Synthesis

Synthesis of Rh-HDL NPs was performed as previously described^14^,^21^. Citrate-stabilized 5nm diameter gold particles (Ted Pella, 15702-5) were incubated with a 5-fold excess of purified human apolipoprotein AI (Apo AI, Meridian Life Sciences, A01236H) at room temperature with gentle shaking for one hour. Ethanol was added to the synthesis at a final volume of 20%. Ethanol contributed from lipids was considered in the 20%. All lipids were re-suspended to 1mM concentration in ethanol. Lipids were added in excess to gold concentration for a final of 250-fold for 1,2-dipalmitoyl-sn-glycero-3-phosphoethanolamine-N-[3-(2-pyridyldithio)propionate] (Avanti Polar Lipids, 870205P), and 250-fold for 1,2-dipalmitoyl-sn-glycero-3-phosphocholine (DPPC, Avanti Polar Lipids, 850355P). For particles containing the rhodamine labeled phospholipid, DPPC was reduced to 200-fold, and a 50-fold excess of 1,2-dioleoyl-sn-glycero-3-phosphoethanolamine-N-(lissamine rhodamine B sulfonyl) (Avanti Polar Lipids, 810157P) was added. Solutions were allowed to incubate overnight at room temperature with gentle shaking.

Nanoparticles were then purified using tangential flow filtration (Spectrum Laboratories) using a mPES MidiKros 50kD filter module (Spectrum Laboratories, D02-E050-05-N). Nanoparticles were sterile-filtered via passage through a 0.2 μm filter (VWR, 28145-501) and then quantified via UV-Vis spectroscopy by measuring the peak absorbance (λ_max_) at ~520 nm (extinction coefficient, ε for 5 nm diameter AuNPs = 9.696 × 10^6^ M^–1^cm^–1^). The size of the resultant conjugates was measured using dynamic light scattering (DLS, Zetasizer Nano ZS, Malvern). Particles were stored at 4 °C and protected from light. The number of rhodamine-labeled phospholipids per HDL NP was calculated by measuring the fluorescence liberated from Rh-HDL NPs at 1nM (as measured by Au concentration), 10 minutes after addition of potassium cyanide (KCN) to 1nM. Rhodamine phospholipids at known concentration were also treated with KCN and fluorescence analyzed to generate a standard curve in order to quantify molar concentration of rhodamine lipid in the 1nM particle sample (thus number of rhodamine lipids per particle of Au).

### Cell Culture

CWR22Rv1 prostate cancer cells (a generous gift from D. Vander Griend Lab) were maintained in RPMI-1640 media (Corning, 10-041-CV) and A375 melanoma cells (CRL-1619, ATCC) were maintained in Dulbecco’s Modification of Eagle’s Medium (DMEM, Corning, 10-013-CV). Both media types were supplemented with 10% FBS (Atlanta Biologicals, S11150H) and 1% penicillin/streptomycin (GE Healthcare Life Sciences, SV30010). Both cells were cultured in 10% CO_2_ at 37 °C in a humidified incubator.

The GFP-SR-B1 plasmid (pEGFP-N1, a generous gift from the Dhe-Paganon Lab) was stably transfected into the A375 melanoma cells using Lipofectamine 2000 (Life Technologies, 11668027). Transfected cells were selected using 500 μg/mL Geneticin (Life Technologies, 10131-027) followed by sorting on a FACSAria cell sorter (BD Biosciences) at the Robert H. Lurie Cancer Center Flow Cytometry Core Facility.

For exosome production, cells were cultured to approximately 70% confluency in five 152 cm^2^ tissue culture plates (Corning, 353025), washed twice with 0.1 μm filtered phosphate buffered saline (PBS), and the media replaced with 20 mL of media containing 10% exosome depleted FBS and 1% penicillin/streptomycin. Exosomes were depleted from FBS via ultracentrifugation in a Beckman Coulter ultracentrifuge at 110,000 × *g* for 18h. Media was also filtered using a 0.1 μm filter unit (VWR, 89220-696).

In a typical experiment for Rh-HDL NP addition, the particles were added on day 1 to media at a concentration of 10 nM total. 48 hours later, additional Rh-HDL NPs were added to a final concentration of 20 nM. 96 hours after initial treatment, the media was pooled from the tissue culture plates and centrifuged at 3000 × *g* for 15 minutes to remove cell debris. Cells were removed from flasks using TrypLE Express (Thermo Fisher, 12604013), counted using a Countess automated cell counter (Life Technologies), and frozen for protein analysis or washed and re-suspended in fresh media for flow cytometry analysis on an LSRFortessa cell analyzer (BD Biosciences).

### Exosome Isolation

For electron microscopy, exosomes were isolated using ExoQuick TC (System Biosciences, EXOTC10A-1) according to manufacturer’s instructions. This was in order to provide a matrix so the exosomes could be embedded for sectioning. For all other assays including size analysis, western blot, and flow cytometry, exosomes were isolated from tissue culture media using ultracentrifugation in a Beckman Coulter ultracentrifuge using a Ti 45 rotor according to the protocol in Thery *et al.*^24^. Briefly, conditioned media was separated into 50 mL aliquots, spun for 30 minutes at 10,000 × *g*, supernatant removed, and supernatant was then spun at 100,000 × *g* for 75 minutes. The pellet was then re-suspended in 100–200 μL 1× PBS filtered through a 0.1 μm filter (VWR). Exosomes from cells treated with Rh-HDL NPs were centrifuged again at 15,800 × *g* in order to pellet the gold nanoparticles and separated into a supernatant and pellet fraction for further analysis. The pellet was re-suspended in 100–200 μL filtered 1× PBS.

### Exosome Characterization

Exosome size was determined using DLS on the Zetasizer Nano ZS (Malvern) using the number function. Nano tracking analysis (NTA) was performed using the NanoSight LM10-HS (Malvern) at the Northwestern University Keck Biophysics Facility. Exosomal protein concentration was determined via BCA Protein Assay (Thermo Scientific, 23227). Gold and rhodamine content was observed using UV-Vis spectroscopy and absorbance readings at 520 nm and 560 nm, respectively.

### Western Blot

Cells were lysed using M-Per Mammalian Protein Extraction Reagent (Thermo Scientific, 78501) and protein concentration determined using BCA assay, as above. 20 μg total cell lysate or exosomes were mixed with 4× Laemmli sample buffer (Bio-Rad, #1610747) containing no reducing agent (CD81 and CD63 only)^24^ or 2-mercaptoethanol (SR-B1, PSMA, and beta actin). Samples were incubated at 95 °C for 5 minutes before being loaded onto a 4–20% glycine polyacrylamide mini gel (Bio-Rad, #4561093S) and electrophoresed for 32 minutes at 200V. Gels were transferred to a polyvinylidene fluoride membrane (Bio-Rad, #1620175) at 60V for 90 minutes then blocked with 5% milk in Tris-buffered saline containing 0.1% Tween-20 (TBS-Tween) for 1 hour. Membranes were then incubated overnight at 4 °C with antibodies listed in Supplementary Table S2, all diluted in 5% milk in TBS-Tween. The following day, membranes were washed with TBS-Tween and then incubated for one hour with secondary antibodies as listed in Supplementary Table S2, all diluted in 5% milk in TBS-Tween. Membranes were then washed with TBS-Tween and developed using the Amersham ECL Western Blotting Detection Reagent (GE Healthcare Life Sciences, RPN2106) and developed on Hyperfilm ECL (GE Healthcare Life Sciences, 28906839) according to the manufacturer’s instructions.

### Electron Microscopy

Exosome pellets obtained from ExoQuick TC precipitation were embedded in agarose for processing and sectioning. Samples were sectioned to a thickness of 50 μm and imaged on a FEI Tecnai Spirit G2 transmission electron microscope at the Northwestern University Center for Advanced Microscopy operating at 120 kV.

### Bead-Associated Flow Cytometry

Bead-assisted flow cytometry was performed using the ExoFlow kit (System Biosciences, EXOFLOW400A-1). Beads were conjugated to biotinylated anti-CD81 (provided with kit), biotinylated anti-rhodamine (Vector Labs, BA-0605), or biotinylated anti-PSMA (BioLegend, 342510). 100 μg of exosomes were added to each reaction and carried out according to manufacturer’s instructions. Flow cytometry was performed on a LSRFortessa Special Order Research Product (SORP) Cell Analyzer custom fitted with low noise Versa Module Europa Peripheral Component Connect Extents (VPX) electronics (BD Biosciences) at the Robert H. Lurie Cancer Center Flow Cytometry Core Facility using the gating strategy recommended in the documentation included with the ExoFlow kit.

### Bead-Free Flow Cytometry From Ultracentrifugation Exosome Isolates

A BD LSRFortessa SORP Cell Analyzer custom fitted with low noise VPX electronics (BD Biosciences) was calibrated for detection of nanoparticles in the <200 nm size range using Megamix-Plus SSC beads (Biocytex, 7803) and methodology defined by the manufacturer (see Supplementary Fig. S5 for detailed gating strategy). 50 μl of exosomes (concentration: 8 ng/μL) from cells treated with Rh-HDL NPs were stained with 2.5 μL APC anti-CD81 antibody (BioLegend, 349510) or 2.5 μL APC anti-CD63 antibody (BioLegend, 353008) for 30 minutes at room temperature. Stained exosomes were diluted to a concentration of 1 ng/μL in 1× PBS before data was acquired on the calibrated analyzer. Data was acquired for 10 minutes at low setting for all samples.

For experiments where pre-isolated exosomes were labeled by Rh-HDL NPs, 50 μl of exosomes (concentration: 8 ng/μL) isolated from untreated A375 cells were incubated with 20 nM Rh-HDL NP for 1 hour at room temperature. Samples were then stained with APC anti-CD81 or APC anti-CD63 as above, and analyzed on the calibrated analyzer as above.

### SR-B1 Knockdown Experiments

GFP-SR-B1 A375 cells were plated in a 24-well plate at a concentration of 5,000 cells per well (a low cell number was used due to the fast growth rate of this line) and allowed to adhere overnight. The second day, 20 pmol control silencer RNA (Life Technologies, AM4611) or siSR-B1 (Wako Chemicals, 299–75001) was transfected to cells in appropriate wells using Lipofectamine RNAiMAX (Life Technologies, 13778030) according to manufacturer’s instructions. 24 hours later, media was removed, cells washed, and media replaced with fresh 0.1 μm filtered DMEM. For experiments testing SR-B1 involvement in cellular processing of Rh-HDL NP into forming exosomes, Rh-HDL NP was added to appropriate wells to a concentration of 20 nM. For experiments testing SR-B1 involvement in labeling of excreted exosomes, no Rh-HDL NP was added at this time. 18 hours later, media was collected, filtered through a 0.2 μm filter to remove dead cells, and a 50 μL aliquot was stained with 2.5 μL APC anti-CD81 antibody for 30 minutes at room temperature. The sample was then diluted 1:30 in filtered PBS and analyzed on a LSRFortessa SORP analyzer calibrated for microparticle analysis as above.

### Exosome Identification in Human Serum by Direct Flow Cytometry

Fresh whole blood was obtained from a healthy adult volunteer. Rh-HDL NP A375 exosomes were added to 100 μL whole blood at varying concentrations including 0, 0.1, 0.5, 1.0, 2.0, 3.0, and 4.0 ng/μL. Additionally, Rh-HDL NP was added to blood in an equivalent concentration (based on gold concentration) as found in the 4.0 ng/μL exosome group. The sample was diluted 1:2 using 1× PBS and depleted of erythrocytes using Histopaque-1077 (Sigma-Aldrich, 10771) according to manufacturer’s instructions. The serum component was centrifuged at 3000 × *g* for 15 minutes to remove any remaining cells. A 50 μL aliquot of supernatant was stained with 2.5 μL of APC anti-CD81 antibody for 30 minutes at room temperature and then diluted 1:400 before being analyzed on a LSRFortessa SORP analyzer calibrated as described in the previous section. Data was acquired for 3 minutes at low setting for all samples. Remaining serum was incubated at 37 °C in a tissue culture incubator and an aliquot was analyzed as above 24 hours later.

### Analysis of Human Serum Exosomes

Human melanoma patient serum was isolated from whole blood obtained from the Interdisciplinary Melanoma Cooperative Group at NYU’s Perlmutter Cancer Center, frozen at −80 °C, and shipped overnight on dry ice to Northwestern University. For western analysis, samples were defrosted and exosomes isolated using ExoQuick (System Biosciences, EXOQ5A-1) according to the manufacturer’s instructions. Isolated exosomes were stored at −80 °C until time of analysis. Exosome concentration was determined via BCA protein assay as above.

For flow analysis, 50 μl of serum was incubated with 20 nM Rh-HDL NP for 1 hour at room temperature. Samples were then stained with APC anti-CD81, diluted 1:500, and data collected for 10 minutes on a calibrated LSRFortessa cell analyzer as described above. Remaining sample was then spun at 15,800 × *g* for 50 minutes, supernatant removed, and pellet resuspended in 10 μL molecular-biology grade water for western analysis as described above.

### Exosome Uptake Experiments

Wild-type A375 melanoma cells were grown in exosome-free media as detailed above. Conditioned media was collected and treated with Vybrant DiO Cell-Labeling Solution (Thermo Fisher, V-22886) according to manufacturer’s instructions. Exosomes were isolated and washed and a portion incubated with 20 nM of Rh-HDL NP for 1 hour at room temperature. An aliquot was then stained with APC anti-CD81 or APC anti-CD63 for 30 minutes, diluted 1:400, and analyzed on the calibrated LSRFortessa cell analyzer as above. Upon verification of exosome presence, 100 μg of exosomes from each group (untreated and Rh-HDL NP treated) were incubated with A375 cells seeded in a 12-well plate at a density of 500,000 cells per well. Cells from individual wells were collected at 2 hours and 24 hours. Cells were washed twice with 1× PBS, removed using TrypLE Express, and re-suspended in 400μL fresh media for flow cytometry using an LSRFortessa cell analyzer.

### Data Analysis

Data analysis of flow cytometry files was performed using FCS Express Version 4 (De Novo Software) and FlowJo Version 4× (FlowJo, LLC). Statistical analysis was performed using Prism software (GraphPad Software). Statistical tests used are indicated in figure legends. All error bars represent standard deviation of the sample.

## Additional Information

**How to cite this article**: Angeloni, N. L. *et al.* Pathways for Modulating Exosome Lipids Identified By High-Density Lipoprotein-Like Nanoparticle Binding to Scavenger Receptor Type B-1. *Sci. Rep.* **6**, 22915; doi: 10.1038/srep22915 (2016).

## Supplementary Material

Supplementary Information
