# Supplementary material for: Pathways for Modulating Exosome Lipids Identified By High-Density Lipoprotein-Like Nanoparticle Binding to Scavenger Receptor Type B-1
Source: Sci Rep. 2016 Mar 11;6:22915. doi: 10.1038/srep22915 (PMC4786789; doi:10.1038/srep22915)
Supplement: Supplementary Information [file srep22915-s1.pdf]

Supplementary Materials:

Pathways for Modulating Exosome Lipids Identified By High-Density Lipoprotein-Like Nanoparticle Binding to Scavenger Receptor Type B-1

Authors: Nicholas L. Angeloni, Kaylin M. McMahon, Suchitra Swaminathan, Michael P. Plebanek, Iman Osman, Olga V. Volpert, and C. Shad Thaxton*

*Correspondence to: C. Shad Thaxton (cthaxton003@md.northwestern.edu)

This file includes:

Figures S1-S11

Tables S1-S2

Supplementary Materials:

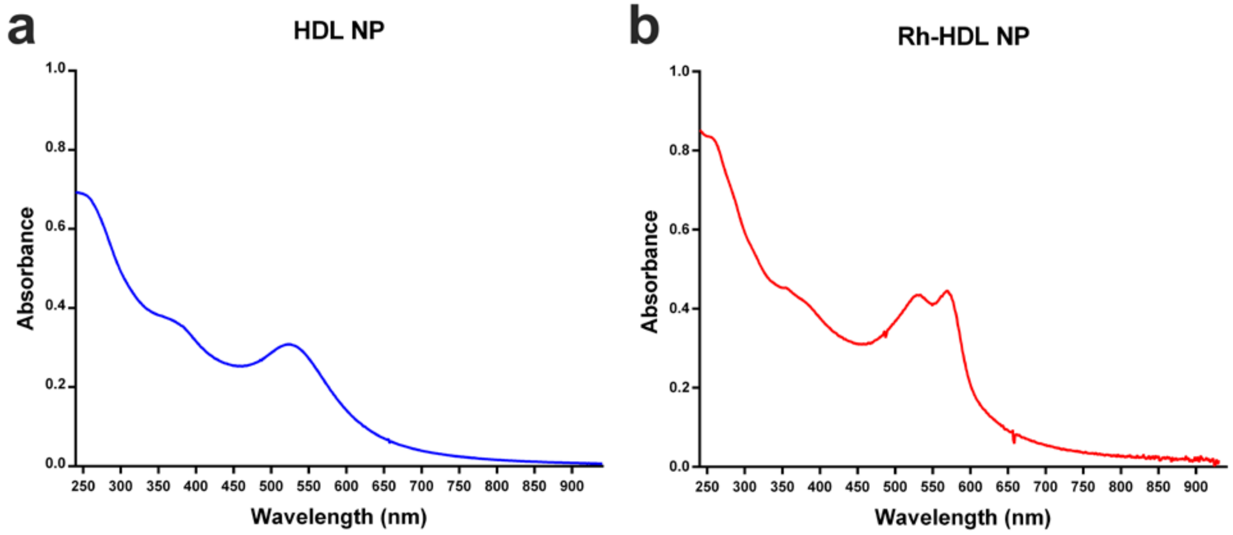

Supplementary Figure S1. (**a**) UV-Vis spectrum of HDL NP showing characteristic surface plasmon for gold nanoparticles at 520 nm. (**b**) UV-Vis spectrum of Rh-HDL NP, demonstrating an additional absorbance peak at 560 nm, corresponding to the rhodamine fluorophore.

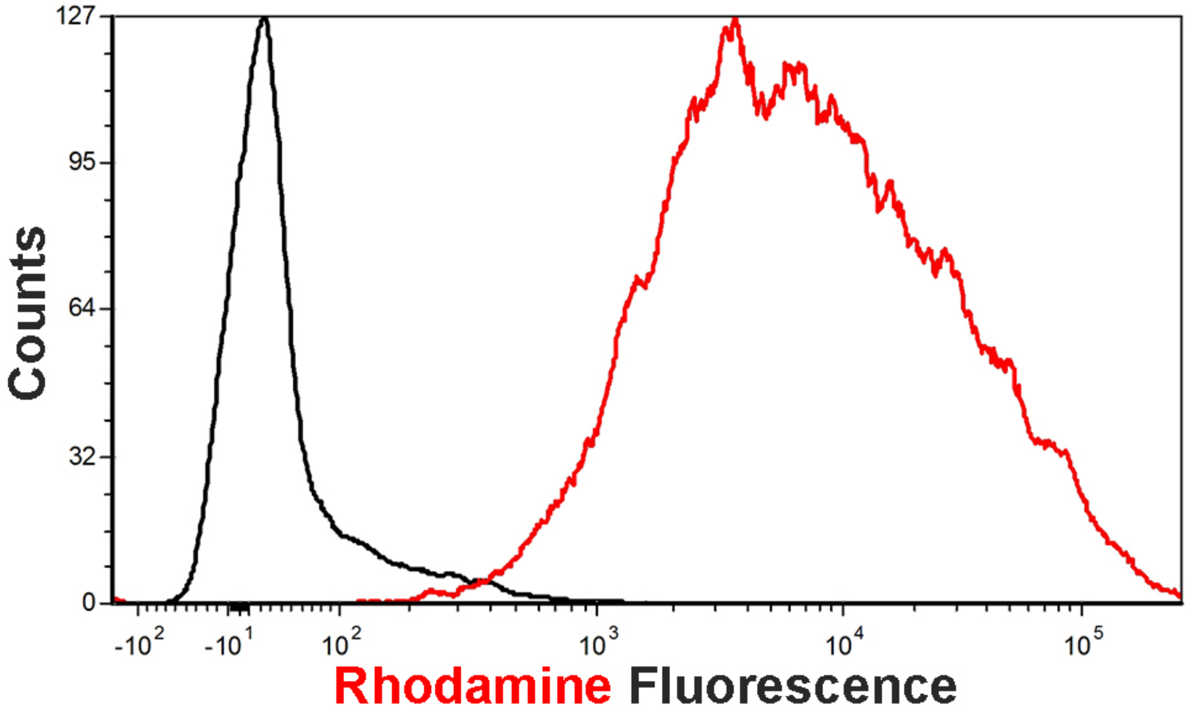

Supplementary Figure S2. Rh-HDL NP uptake by CWR22Rv1 cells. Histogram of rhodamine fluorescence from untreated cells (black) and cells incubated with 20nM Rh-HDL NP (red).

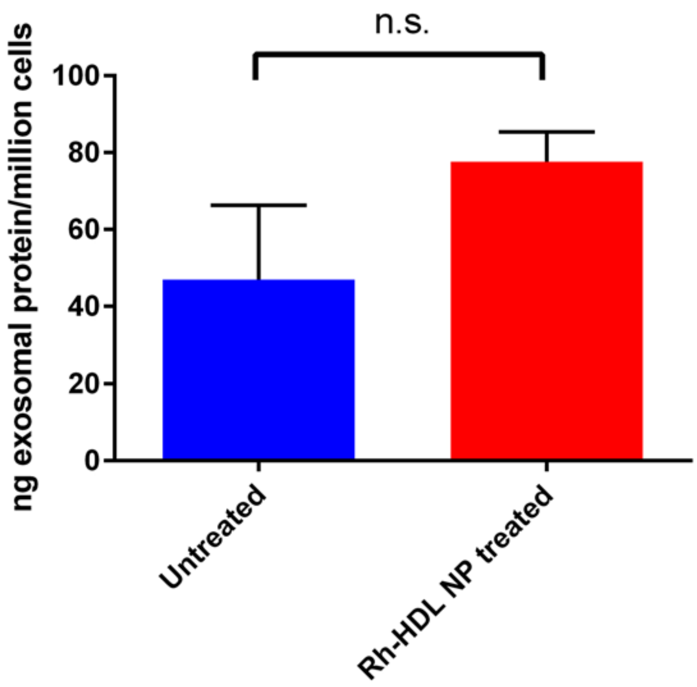

Supplementary Figure S3. Exosome production by CWR22Rv1 cells. Untreated cells and Rh-HDL NP treated cells do not produce statistically different amounts of exosomal protein (P = 0.22).

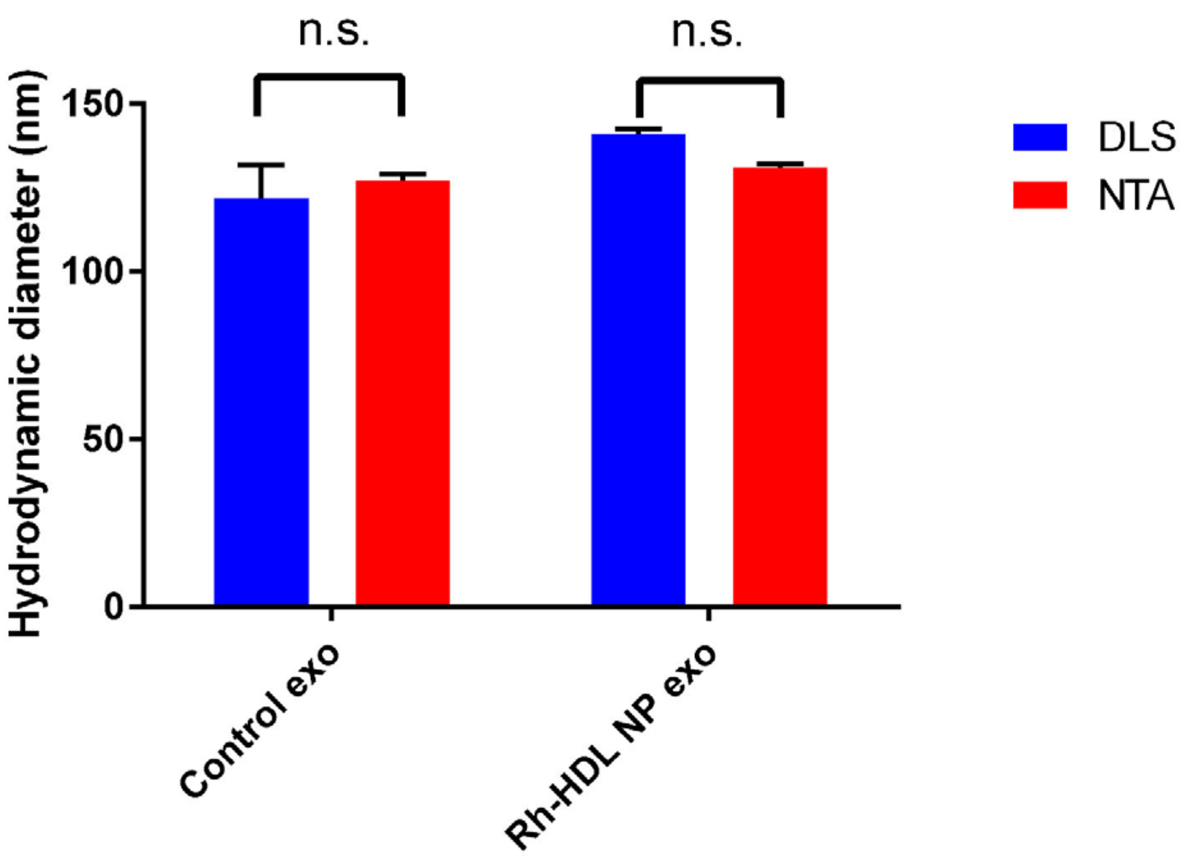

Supplementary Figure S4. Exosome size measured by DLS and NTA. The hydrodynamic diameter of exosomes from untreated control (left) and Rh-HDL NP treated cells (right) was determined by DLS (blue bar) and NTA (red bar) and found not to be statistically different ($p > 0.9999$).

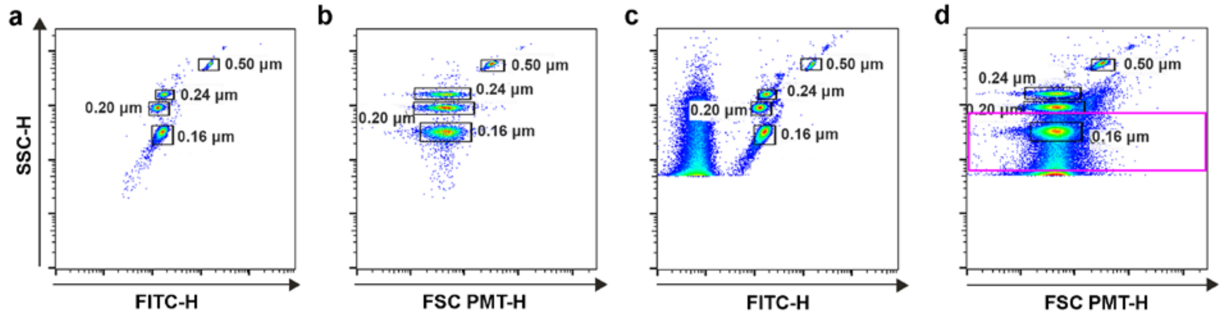

Supplementary Figure S5. Gating strategy for analysis of bead-free exosomes. The Megamix+ SSC beads were run on the BD LSRFortessa SORP with low noise VPX electronics. As indicated in Megamix+ SSC protocol (**a**) the bead system was first analyzed using threshold on FITC, eliminating non-fluorescent background noise. (**b**) The corresponding events displayed on side scatter height (SSC-H) versus forward scatter photomultiplier height (FSC PMT-H) plot with FITC threshold. (**c**) The bead system was then analyzed in a second step using SSC as threshold. (**d**) The corresponding events displayed on SSC-H versus FSC PMT-H plot with SSC threshold for evaluation of SSC resolution and background. A clear separation of the beads (0.16 µm, 0.20 µm, 0.24 µm and 0.50 µm) on SSC are observed with low background and standardized minimum threshold was setup by running ultra-clean water (0.1 µm filtered) as defined in the protocol. The final minimal threshold on scatter was then setup by running ultra-clean water (0.1 µm filtered) and progressively reducing threshold conditions until total events pass over 50% of the maximum speed of analysis authorized for our instrument. We then went back to a slightly higher value of SSC threshold to get back below 50% of the maximum events, thus validating and setting up the instrument for small particle analysis. All events analyzed were in a gate in the region less than 0.20 µm (pink gate, **d**).

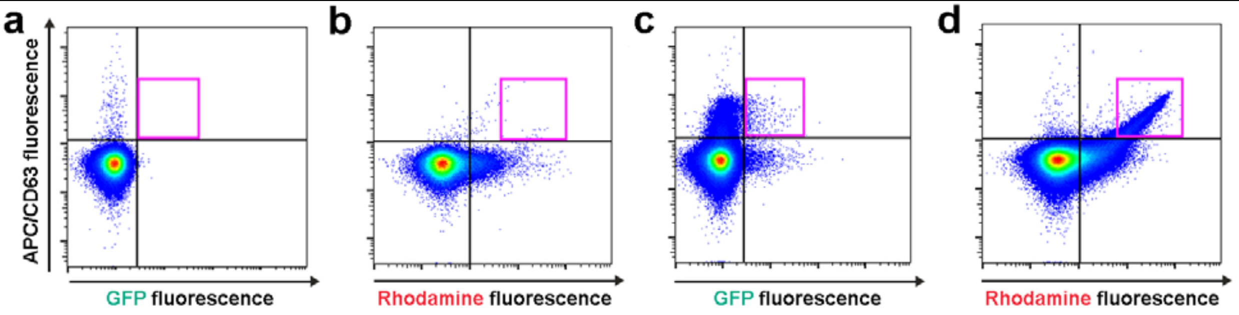

Supplementary Figure S6. CWR22Rv1 exosomes can be detected via CD63 stain. Direct flow cytometry of: (**a**) APC anti-CD63 antibody alone (control) (**b**) CD63 antibody and Rh-HDL NP (control) (**c**) CD63+/ GFP+ exosomes (pink box), and (**d**) CD63+/ Rh+ exosomes (pink box).

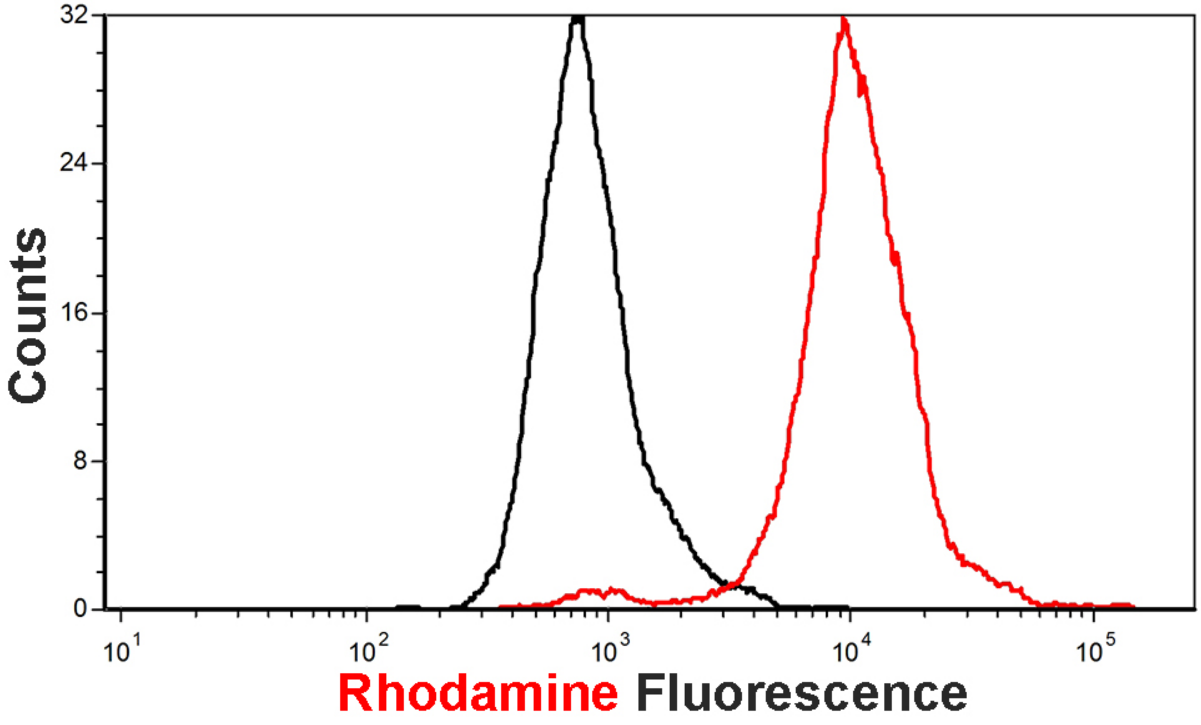

Supplementary Figure S7. Rh-HDL NP uptake by A375 cells. Histogram of rhodamine fluorescence from untreated cells (black) and cells incubated with 20nM Rh-HDL NP (red).

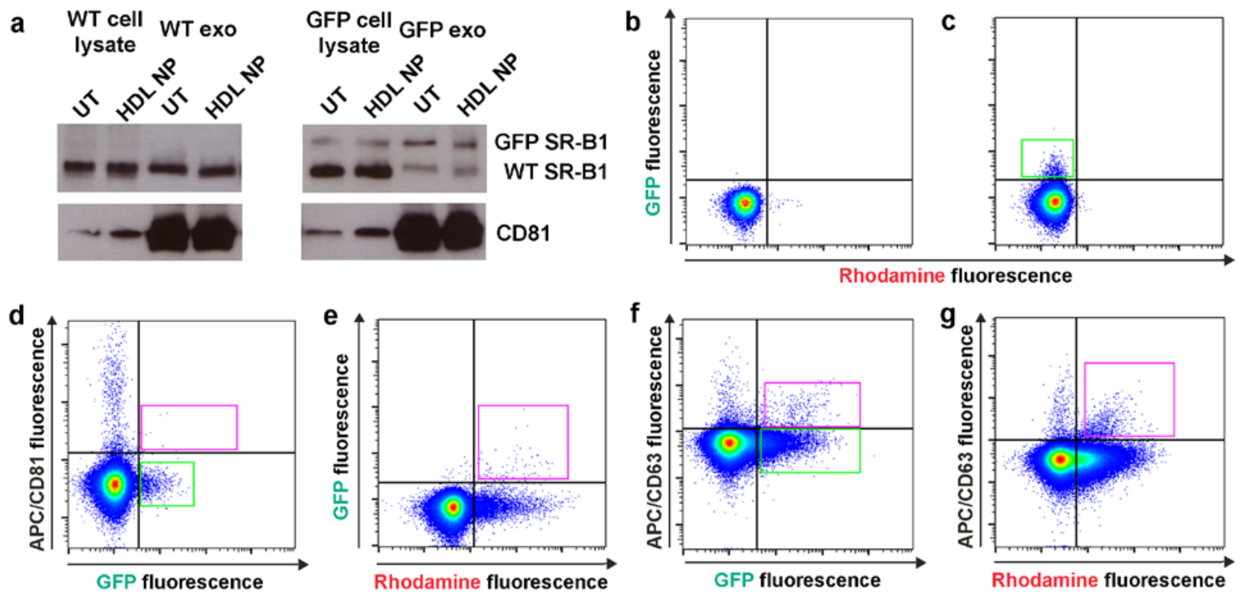

Supplementary Figure S8. GFP-SR-B1 incorporation and Rh-HDL NP labeling of exosomes. (**a**) Western blot expression of SR-B1 and CD81 in wild-type and GFP-SR-B1 A375 melanoma cells and derived exosomes. (**b**) Wild-type A375 exosomes isolated by ultracentrifugation. (**c**) GFP-SR-B1 A375 exosomes isolated by ultracentrifugation showing GFP expression (green box). (**d**) GFP^+ particles in exosomes from GFP-SR-B1 A375 cells (green box) stained for CD81 (co-labeling in pink box). (**e**) GFP^+ particles in exosome prep from GFP-SR-B1 A375 cells treated with Rh-HDL NP are Rh^+ (pink box). (**f**) GFP^+ particles in exosomes from GFP-SR-B1 A375 cells (green box) stained for CD63 (co-labeling in pink box). (**g**) Rh^+ particles in exosomes from GFP-SR-B1 A375 cells stained for CD63 (co-labeling in pink box).

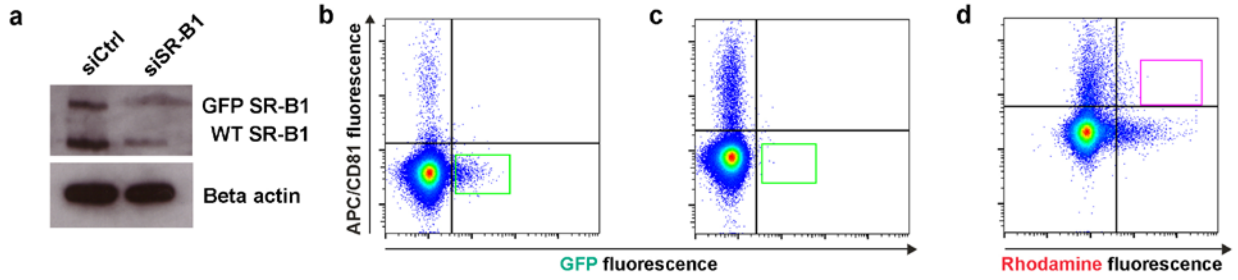

Supplementary Figure S9. Knockdown of SR-B1 by siRNA. (**a**) Western blot of siCtrl (left) and siSR-B1 (right) treated GFP-SR-B1 A375 cells. (**b**) Conditioned media from siCtrl treated GFP-SR-B1 A375 cells and (**c**) A375 cells treated with siSR-B1. (**d**) Culture media treated with APC anti-CD81 antibody and Rh-HDL NP.

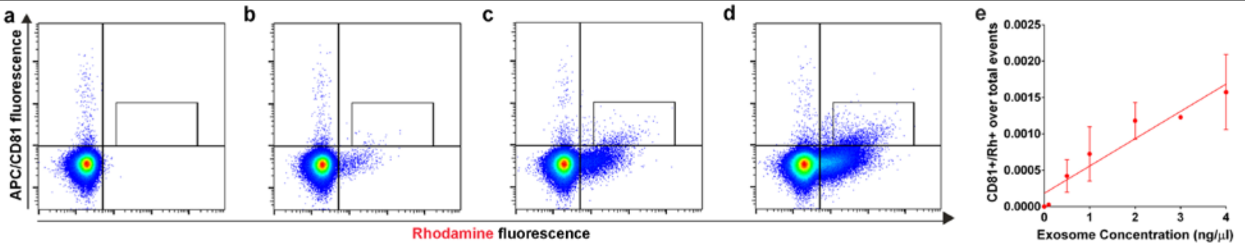

Supplementary Figure S10. Detection of A375 exosomes labeled with Rh-HDL NP spiked into human blood at T = 0. (**a**) Serum with APC anti-CD81 and no exosomes. (**b**) 0.1 ng/µL added exosomes. (**c**) 1.0 ng/ µL added exosomes. (**d**) 4.0 ng/µL added exosomes. (**e**) Quantification of gated events normalized to all events plotted against exosome concentration (r^2 = 0.8144)

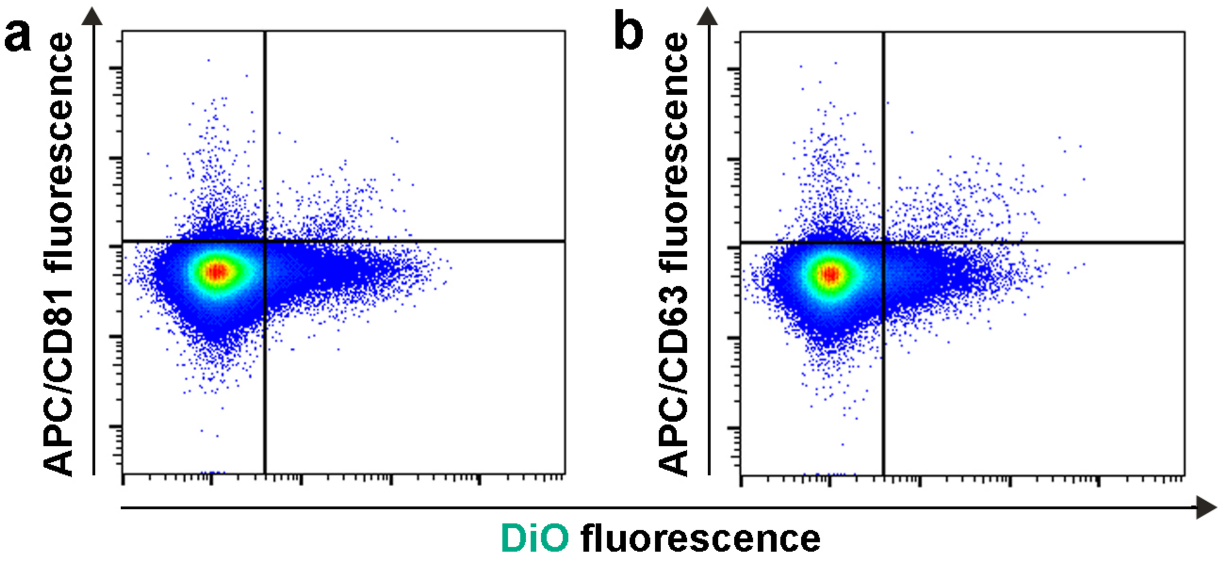

Supplementary Figure S11. DiO labels CD81 and CD63 exosomes. DiO stained exosomes labeled with (**a**) anti-CD81 or (**b**) anti-CD63 antibody.

Supplementary Table S1. Physical characterization data for Rh-HDL NPs.

	HDL NP	Rh-HDL NP
Size (nm)	13.64 ± 1.23	12.85 ± 0.44
UV-Vis λ^{max} (nM)	519	517
Zeta Potential (mV)	-41.6 ± 0.71	-37.9 ± 2.12
Rhodamine lipids / particle	0	17

Supplementary Table S2. Primary and secondary antibodies and concentrations used for immunoblotting.

Primary Antibody	Dilution	Secondary Antibody	Dilution
CD81 (Santa Cruz, sc-23962)	1:250	Goat anti-mouse (Bio-Rad, 170-6516)	1:2000
CD63 (Novus, NB100-77913)	1:1000	Goat anti-mouse (Bio-Rad, 170-6516)	1:2000
SR-B1 (Abcam, ab52629)	1:1000	Goat anti-rabbit (Bio-Rad, 170-6515)	1:2000
Beta actin (Cell Signaling, 4970C)	1:1000	Goat anti-rabbit (Bio-Rad, 170-6515)	1:2000
Y-PSMA (Abcam, ab19071)	1:1000	Goat anti-mouse (Bio-Rad, 170-6516)	1:2000
